# The Influence of Work-Related Chronic Stress on the Regulation of Emotion and on Functional Connectivity in the Brain

**DOI:** 10.1371/journal.pone.0104550

**Published:** 2014-09-03

**Authors:** Armita Golkar, Emilia Johansson, Maki Kasahara, Walter Osika, Aleksander Perski, Ivanka Savic

**Affiliations:** 1 Department of Clinical Neuroscience, Karolinska Institute, Stockholm, Sweden; 2 Center for Social Sustainability, Department of Neurobiology, Care Sciences and Society, Karolinska institute, Stockholm, Sweden; 3 Stress Research Institute, Stockholm University, Stockholm, Sweden; 4 Department of Women's and children's health, and Neurology Clinic, Karolinska Institute and Hospital, Stockholm, Sweden; Max Planck Institute for Human Cognitive and Brain Sciences, Germany

## Abstract

Despite mounting reports about the negative effects of chronic occupational stress on cognitive and emotional functions, the underlying mechanisms are unknown. Recent findings from structural MRI raise the question whether this condition could be associated with a functional uncoupling of the limbic networks and an impaired modulation of emotional stress. To address this, 40 subjects suffering from burnout symptoms attributed to chronic occupational stress and 70 controls were investigated using resting state functional MRI. The participants' ability to up- regulate, down-regulate, and maintain emotion was evaluated by recording their acoustic startle response while viewing neutral and negatively loaded images. Functional connectivity was calculated from amygdala seed regions, using explorative linear correlation analysis. Stressed subjects were less capable of down-regulating negative emotion, but had normal acoustic startle responses when asked to up-regulate or maintain emotion and when no regulation was required. The functional connectivity between the amygdala and the anterior cingulate cortex correlated with the ability to down-regulate negative emotion. This connectivity was significantly weaker in the burnout group, as was the amygdala connectivity with the dorsolateral prefrontal cortex and the motor cortex, whereas connectivity from the amygdala to the cerebellum and the insular cortex were stronger. In subjects suffering from chronic occupational stress, the functional couplings within the emotion- and stress-processing limbic networks seem to be altered, and associated with a reduced ability to down-regulate the response to emotional stress, providing a biological substrate for a further facilitation of the stress condition.

## Introduction

Stress is common and hard to avoid. When stress becomes chronic, it may have negative effects on cognitive functioning and even lead to psychiatric conditions such as anxiety and depression [1]–[2]. In recent years, the mounting reports about occupational stress and the substantial costs for society that are associated with it, mainly due to impaired mental health, have been gaining more attention [3]. These worldwide reports signal the pressing need for scientific investigations of the underlying pathophysiological mechanisms.

### Cognitive and emotional dysfunctions attributed to occupational stress – ‘the burnout syndrome’

Occupational ‘burnout’ is characterized by stress-related symptoms among otherwise healthy and high-performing persons who report that they have not experienced any major negative life events [4]–[8]. The described symptoms are attributed to occupational stress. They are stereotyped, and include memory and concentration problems, sleeplessness, diffuse aches, profound fatigue, irritability, anxiety, and a feeling of being emotionally drained. The underlying mechanisms are largely unknown. Measurements of cortisol levels after awakening in these subjects have hitherto yielded inconclusive results [9], with reports of normal [10]–[11], reduced [12]–[15], and elevated levels [16]–[18]. Recent data from brain imaging studies, although still limited, suggest, however, that burnout from occupational stress is associated with an affection of the limbic structures, the amygdala, and the mesial prefrontal cortex (mPFC), in particular [19]–[23]. These initial findings call for further research, considering the amygdala's key role in evoking stress responses [24]–[25] and considering that the regulation of stress responses during emotional conflict is processed via functional connectivity between the amygdala and the mPFC and anterior cingulate cortex (ACC) [26]–[30]. During the cognitive reappraisal of emotion, it has, for example, been demonstrated that the activity of the amygdala is down-regulated (measured as change in BOLD signal), whereas the activity in portions of the lateral and medial prefrontal cortex is upregulated [31]–[36]. Moreover, it was recently shown that the ability to cognitively down- regulate negative emotion was severely jeopardized after stress exposure [37]. It is, thus, possible that subjects reporting cognitive and emotional dysfunction due to chronic occupational stress could have an impaired ability to modulate emotional stress and emotionally stressful stimuli, rendering them less apt to cope with psychosocial stress. Furthermore, in these individuals, the amygdala connectivity with the mPFC and ACC, and perhaps also to the hippocampus and the insular cortex, could have undergone alterations. Such changes could constitute a stress vulnerability factor, or be a consequence of prolonged occupational stress. Both scenarios would be in line with our previous observations of structural and neuroreceptor changes along the limbic circuits in affected subjects [20]–[23]. These limbic changes, and their apparent overlap with the networks that are reported to be involved in emotional regulation led us to design a combined behavioral and MR study to test two specific hypothesis: (1) That subjects suffering from occupational stress have an impaired ability to modulate stressful emotions; and (2) That these subjects show altered amygdala functional connectivity. To test these, forty subjects with occupational burnout along with seventy unstressed healthy controls were investigated using a cognitive emotion regulation task as well as resting state fMRI.

### Emotion regulation and the acoustic startle reflex

In order to assess emotion regulation, we measured the magnitude of the fear-potentiated startle reflex, which is a highly conserved, fast defensive reflex that consists of a series of muscular contractions and is mediated by a well-characterized neural circuitry [38]–[39]. In humans, this reflex can be elicited by a sudden and intense auditory stimulus (acoustic startle probe). The amplitude of this reflex is measured through facial electromyography (EMG), [40], and is potentiated when the individual is in an aversive or fearful state [41]. The startle reflex is a reliable and well-validated measure of emotion modulation [40] and has previously been successfully used as an index of cognitive emotion regulation in a healthy population [42]–[43].

In the present study the acoustic startle reflex was measured to investigate possible group differences in emotional reactions to negative visual stimuli and in the ability to regulate negative emotion. We also measured functional resting state connectivity from the amygdala, paying special attention to the connectivity to other nodes of the limbic system and to the mPFC and ACC in particular. In addition, a possible association between the ability to modulate emotion and the resting state functional connectivity was tested with linear regression analysis.

## Methods

### Participants

Forty right-handed [44] subjects (27 females; age 38±6 years, range 19–46 years; education 17±3 years), who had been diagnosed as having a ‘reaction to severe stress and an adjustment disorder’ according to the International Classification of Diseases (ICD-10, F43), were recruited from the Stress Research Institute at Stockholm University. The selection was limited to subjects who attributed their illness to prolonged work-related stress, after working 60 to 70 hours per week continuously over several years prior to the onset of symptoms. Inclusion criteria consisted of a characteristic symptom course of sleeplessness, diffuse aches, palpitations and fatigue, a subsequent onset of irritability, anxiety, memory and concentration problems, feeling of depersonalization, and reduced work capacity (confirmed by the employers) [8],[19]. All of the subjects attributed their symptoms to chronic stress and had no other known etiology for their distress.

Subjects were also required to have had a symptom duration of at least one year (their histories of stress-related burnout symptoms ranged from 1.5 to 3.5 years), to have been on sick leave (≥50%) for stress-related symptoms for a minimum of 6 months before entering the study, and to have an average stress-burnout score of *≥3.0* on the Maslach Stress-Burnout Inventory – General Survey (MBI-GS) [45]. This 7-point rating scale, ranging from 0 (never) to 6 (daily), consists of three subscales: exhaustion (five items), cynicism (five items) and lack of professional efficacy (six items). When rating perceived stress, subjects were asked to take into consideration the last six months, and not only the actual time-point. The average scores for Scandinavian populations are around 2 for MBI-GS [4],[46].

Subjects were excluded if they had a previous history of psychosis, personality disorder, major or bipolar depression, alcohol or substance abuse, chronic fatigue, chronic pain, fibromyalgia, or neurological or endocrine disease. Those who had experienced prominent stress factors in their private life or a major traumatic event at any time in their life, including sexual abuse, were also excluded. No daily medication was allowed during the two months prior to the study, except contraceptives. According to a review of their pharmacological treatment histories, none of them had taken drugs that are known to affect brain structure (e.g., psychopharmaca). Subjects who were sleep deprived the night before the scan/testing procedures were rescheduled, in order to exclude the acute effects of sleep deprivation.

Seventy healthy, right-handed, non-smoking volunteers (45 females; age 33±6 years, range 24–45 years; education 17±3 years) with no history of chronic stress or heredity for neuropsychiatric disorders comprised the control group. The patient and control groups had similar gender distributions, and both were predominately female to accord with the female-dominated epidemiology of the condition studied [4].

The two groups were matched for socioeconomic status assessed on the basis of years of education, type of occupation, and organizational position (employee, middle management, supervisor). The study was approved by the Ethics Committee at the Karolinska Institute and written informed consent was received from each participant.

Before the interview, participants completed questionnaires in order to evaluate their stress symptoms and assess their previous life events [47]. In addition, the occurrence of major life events among the subjects was assessed through a clinical psychiatric interview based on the non-work-related items of the Holmes and Rahe Scale [48]. The participants were asked to answer yes or no to whether they had experienced any non-work- related stressful life events (e.g., death of a relative or spouse, recent divorce, forced family relocation). Subjects were excluded if they answered positively to having experienced such an event in their lives. Patients also received a medical screening (physical examination, test of thyroid and liver function). A structured interview, the Swedish version of the Mini-International Neuropsychiatric Interview, MINI [49] was performed, along with a test for depression using the Montgomery-Asberg Depression Rating Scale [50]. Although some subjects had high scores in the MADRS they did not fulfill the MINI criteria for depression, and were therefore not excluded.

Out of the participants who matched the inclusion criteria, 8 subjects with occupational burnout and 9 controls failed to display a startle response to the probe. The results from the emotion regulation experiment and the correlation analyses with fMRI are therefore based on data from the remaining 32 subjects with occupational burnout (20 females; mean age = 37.6 years, SD = 6.5) and 61 controls (33 females; mean age = 30.9 years, SD = 6.7), whereas the analysis of resting state amygdala connectivity is based on the entire study group.

### Salivary cortisol

Salivary cortisol was sampled according to a previously established protocol [51]. Saliva sampling was chosen because the method is simple, non-invasive, and non- stressful; the samples are shown to readily reflect the levels of the free fraction of cortisol in plasma [52]. Participants were instructed carefully on how to collect their own salivary samples. Samples were collected seven times on an ordinary weekday using Salivette cotton rolls (Sarstedt, Rommelsdorf, Germany), which participants were instructed to place in the mouth for 2 minutes. The first sample was collected immediately upon awakening in the morning, irrespective of time. The second sample was collected 15 minutes later, before eating or brushing teeth, and the third sample was collected 15 minutes after that. The fourth sample was collected around noontime, before lunch. The fifth sample was collected at about 3 p.m., the sixth at 8 p.m., and the seventh at bedtime, after having rested in bed for 15 minutes, before falling asleep. The samples were frozen (−18°C) until analyzed. The levels of salivary cortisol were measured with radioimmunoassay using the Spectria (^125^I) coated tubes radioimmunoassay kit (Orion Diagnostica, FIN-02101 Espoo, Finland). The within-assay coefficients of variation ranged from 0.8 to 0.9, and those between assays never exceeded 10 percent. All samples from each group were analyzed simultaneously in duplicate.

### Emotion regulation task

Before the experiment, the participants were given written as well as verbal explanations of the task and instructions. Participants were informed that they would receive three different instructions during the experiment and that these instructions would be symbolically represented by three different arrows: (1) an upward arrow indicated that the participant should make an effort to reinforce the feelings that are elicited by the picture, so that he/she experiences the image as more emotionally charged (“up-regulate”); (2) a horizontal arrow indicated that the participant should focus on the feeling the picture elicits, without trying to manipulate the emotion (“maintain”); and (3) a downward arrow indicated that the participant should make an effort to down-regulate the feelings that the picture elicits, so that he/she experiences the image as less emotionally charged, or as neutral as possible (“down-regulate”). Participants were thoroughly informed of the importance of following the instructions during the experiment and not distracting themselves from their feelings by thinking of something else or by looking away from the image or closing their eyes. The subjects were however free to choose the strategy to regulate their emotion.

The experiment began with a practice session during which the participants were first subjected to the auditory startle probe six times to allow for habituation to the sound. This was followed by twelve practice trials that mirrored the experimental procedure. After the practice session, the participants were asked to describe the strategy they had used to regulate emotion. None of the participants reported that they were confused about how to adopt a reappraisal strategy for the neutral and negative trials before or after completing the experimental task.

**Figure 1:**
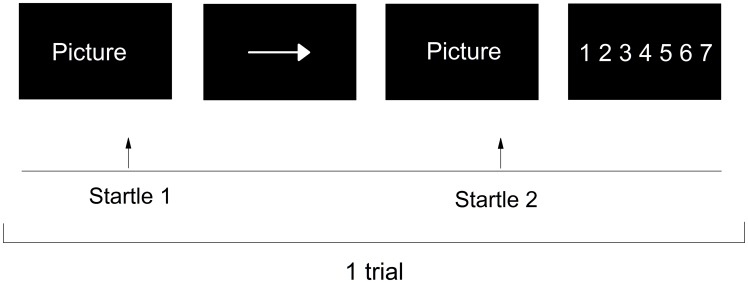
Overview of one experimental trial with the maintain instruction. Participants were presented with a picture, which was replaced by an instruction cue. For negative picture trials, this cue indicated whether the participants' task was to maintain (horizontal arrow), down- regulate (downward arrow) or enhance (upward arrow) their emotional response. Immediately following the instruction cue, participants implemented the regulation instruction while being exposed to the same picture again. Lastly, participants rated how well they managed to implement the regulation instruction on a scale of 1–7.

An example of an experimental trial is shown in Figure 1. During each trial, the participant was presented with a picture for 5 s, which was then replaced by an instruction cue for 1 s. For negative pictures, participants were instructed to suppress (down-regulate), enhance (up-regulate) or maintain their emotional response. Based on previous work [42] and to avoid confusion due to ambiguous instructions (e.g., to suppress emotional reactions to neutral pictures), neutral pictures were only coupled with the instruction to maintain the emotional response. Immediately following the instruction cue, the same picture was presented again for 5 s, during which time the participants carried out the regulation instruction. During each trial, startle probes were presented 3 s after picture onset during the first (pre-instruction) and the second (post-instruction) picture-viewing phases. Lastly, the participants were given 4 s to rate on a scale of 1–7 how well they had managed to carry out the instructions. Between each trial, a fixation cross was presented for 4–6 s (mean 5 s). Each trial lasted for 20 s. There were 60 trials, and the entire testing session lasted approximately twenty minutes, with a 15-second pause after the first 30 trials. The presentation of pictures was synchronized with the monitor's refresh rate and presented with the software Presentation (Neurobehavioural systems, www.neurobs.com).

### Material

We selected three sets of 15 negative pictures and one set of 15 neutral pictures from the International Affective Picture Set (IAPS) [53]. Each of the three sets of negative pictures was assigned to one of the three task instructions (maintain, down-regulate, up-regulate), and this assignment was counterbalanced between participants (male and female controls, and patients). Pictures were selected to match valence and arousal scores of pictures used in a similar report [54].

### Electromyographic recordings: response definition and data reduction

The eye-blink component of the startle response was measured through electromyographic (EMG) recordings of the left orbicularis oculi muscle using two miniature Ag/AgCl electrodes prepared with electrolyte gel. A third ground electrode was placed behind the left ear over the mastoid. Startle probes were 50-ms bursts of approximately 95-db[A] white noise with a near instantaneous rise time (<1 ms), delivered through sound-proof headphones (Bose AE21, Bose Co. Framingham, Massachusetts). The raw EMG signal was amplified and filtered through a 28–50 Hz bandpass filter, rectified and integrated with a time constant of 20 ms. Startle eye-blink magnitude (microvolts) was measured as the amplitude from onset to peak, and trials with excessive baseline activity or recording artifacts were rejected. To assess initial, unaltered startle responses, pre-instruction (Startle 1) startle scores for negative and neutral images were normalized using z-standardization to ensure that all participants contributed equally to the group means, as has been described previously [55]–[56]. The z-score calculation is a within-individual normalization, resulting in a distribution with an overall mean of 0 and a standard deviation of 1 for each participant. To assess the regulation of the startle response according to instruction, for each participant, we calculated the change in startle response by subtracting the raw startle 1 response from the raw startle 2 response separately for each instruction (maintain neutral, maintain negative, down-regulate negative, up-regulate negative) of the task. This way, we defined emotion regulation ability as the magnitude during emotion regulation controlling for baseline levels before the regulation cues.

### Statistical analyses

Initial startle reactions were assessed in a 2×2 repeated measures analysis of variance (ANOVA) with Valence (Negative, Neutral) as a within-subjects variable and Group (Burnout, Control) as a between subjects variable. To test the hypothesis that burnout patients would differ from controls in their startle responses during down-regulation of negative emotion, we ran a 2×2 repeated measures ANOVA with Instruction (Down-regulate, Maintain) as the within-subject variable and Group (Burnout, Control) as the between-subject variable. As a control, we similarly assessed whether there were any group differences in startle responses during up-regulation of negative emotions in a 2 (Up-regulate, Maintain)×2 (Burnout, Control) repeated-measures ANOVA. Possible group difference in salivary cortisol levels was tested with a repeated measure ANOVA (p<.05).

### fMRI data acquisition

MR experiments were carried out on a separate day to avoid contamination by possible effects of the emotional regulation tasks. Functional MRI time series data were collected from all of the participants at rest over 8 minutes in a 3 Tesla MR scanner Discovery 750 (GE Healthcare), using a 32-channel head coil. Resting fMRI blood oxygenation-level dependent (BOLD) data were acquired in a standard gradient echo-planar-imaging (EPI) acquisition, TR = 2.5 s, TE = 30 ms, flip angle = 90°, resolution = 3×3×3 mm, whole-head coverage. The participants were asked to lie with their eyes closed, to think of nothing in particular, and not to fall asleep. Structural brain images were acquired using a T1-weighted 3D brain imaging volume imaging sequence with whole-head coverage, TR = 7.91 s, TE = 3.06 s, flip angle = 12°, and resolution 1×1×1 mm. These structural images were used to aid the registration of the functional data into a common standard brain coordinate system (MNI152).

### Seed region analysis

Seed region analysis is based on calculating cross-correlation coefficients of the time series in a particular seed region-of-interest (ROI) with all other voxels in the brain, which reveals the strength of functional connectivity with respect to this seed region [57]. The seed regions consisted of the right and left amygdala, and were delineated with the guidance of the WFU-pick atlas, and after adaptation to the gray matter template of our own population. The MNI coordinates for the amygdala seeds where (sphere of 5 mm radius, co-ordinate −22, −7 −19, and 22 −7 −19); the seed regions covered the amygdala, with the exception of the most medial 2 mm of the basomedial amygdala, which was excluded to avoid the susceptibility artifact that was detected in some subjects.

Given the amygdala's pivotal role in stress perception, we first evaluated whether and how the functional connectivity from the amygdala seeds differed between patients and controls. We then used multiple regression analysis to investigate whether the degree of perceived stress interacted with the pattern of connectivity from the amygdala seed. Spatial preprocessing and statistical analysis of functional images were performed using SPM8 (Welcome Department of Cognitive Neurology). Functional images were slice-timed and realigned, and then registered to structural T1 SPGR (spoiled gradient) images for each subject. Next, the individual T1 SPGR images were segmented into gray matter, white matter, and cerebrospinal fluid, and the gray matter image was used to determine the parameters of normalization for the standard Montreal Neurological Institute gray matter template. The spatial parameters were then applied to the slice-timed and realigned functional volumes that were finally resampled to 2×2×2 mm voxels and smoothed with a 6-mm full-width at half-maximum kernel. Each voxel's time series was corrected for noise using standard resting-state low-pass filtering with a cut-off frequency of 0.1 Hz. In addition, voxel-wise multidimensional regression analysis was employed in a standardized manner to remove artifacts due to motion and changes in ventricle and white matter signals. This was done by adding six movement regressors obtained from rigid-body head motion correction (SPM 8 statistical package). Segmented WM (white matte) and CSF (cerebro spinal fluid) were used as ROI for correction of signals from non-gray matter tissue. To ensure that signals from WM and CSF ROIs did not contain signals from gray matter, these ROIs were superimposed on the individual EPIs and, when needed, adapted to the respective subject, based on intensity differences between white matter, gray matter, and ventricular regions. Global signal correction was not employed, as it has been reported that regression against the global signal may artificially introduce anticorrelations into fMRI data sets [58]. For each subject, the average fMRI time course within the seed region was used as the regressor of interest. Individual time series in each seed region were extracted with MarsBar toolbox (http://marsbar.sourceforge.net/). Each subject's seed region time course was then regressed voxel-wise against the subject's fMRI time course using the entire brain as search space. The t-values of the corresponding regression coefficients at each voxel were used as each subject's connectivity map.

### Statistical analysis

Group comparisons between stressed subjects and healthy controls were carried out in SPM8 using one-way ANOVA, with p<.001 voxel threshold, FWE corrected at cluster level, p<.05, and controlling for age and gender, which were used as nuisance covariates.

## Results

### Demographics

No group differences were found with regard to education or gender distribution. The controls were younger than the stressed subjects (Table 1). The subjects with occupational burnout scored significantly higher on the MBI-GS scale (3.8±0.8 vs. 2.5±0.7 p = .001; F = 64.3) as well as on the MADRS (16.8±5.5 vs. 3.8±3.8; p = .001, F = 206.8) (Table 1). However, no group difference was detected in cortisol levels (p = .56, F = .08).

**Table 1:** Demographics.

	Stressed subjects (n = 40)	Controls (n = 70)	P and F values
Age (years)	38.2±6.8	33.2±5.8	p = 0.00 F = 17.4
Education (years)	16.9±3.4	16.8±2.9	p = 0.88 F = 0.22
MBI- GS (score)	3.8±0.8	2.5±0.7	p = 0.00 F = 64.3
• exhaustion	4.4±1.1	1.2±0.8	p = 0.00 F = 269.0
• cynicism	3.3±1.3	1.3±1.0	p = 0.00 F = 75.6
MADRS (score)	16.8±5.5	3.8±3.8	p = 0.00 F = 206.8
Cortisol sample 1	15.8±12.6	5.7±3.9	p = 0.86 F = 0.33
Cortisol sample 2	24.2±15.7	15.2±8.5	p = 0.78 F = 0.08
Cortisol sample 3	22.9±14.9	20.3±12.2	p = 0.17 F = 1.92
Cortisol sample 4	8.8±10.0	23.1±16.2	p = 0.96 F = 0.00
Cortisol sample 5	5.8±3.5	9.7±13.9	p = 0.73 F = 0.12
Cortisol sample 6	3.6±4.3	3.7±4.9	p = 0.92 F = 0.01
Cortisol sample 7	4.2±10.8	2.6±3.9	p = 0.33 F = 0.86

Age and education are expressed in years; MBI-GS is a questionnaire to score perceived work-related stress. Raw 3 indicates the mean total score, raw 4–5 the sub-scores for the exhaustion and cynicism. MADRS = Montgomery Asberg Depression Scale. There was no overall group difference in cortisol levels (p = 0.56; F = 0.08, repeated measure ANOVA). Time of the day for cortisol samples: *Sample 1*: 06.30–07.30; *Sample 2*: 15 minutes after sample 1; *Sample 3*: 30 minutes after sample 1; *Sample 4*: 12.00–13.00; *Sample 5*: 15.00–16.00; *Sample 6*: 20.00–21.00. *Sample 7*: 22.30–23.30.

### Emotion regulation task

To verify that the data could be collapsed across female and male controls, we first confirmed that there was no significant difference between female and male controls in their initial startle response to negative and neutral pictures (Valence×Group interaction: F(1,30) = .13, p = .72). Furthermore, no sex differences were found regarding the startle response to negative and neutral pictures across instructions (Instruction×Group interaction: F (2,60) = 2.23, p = .12). Because no significant differences were detected between male and female controls, all of the comparisons with the subjects with burnout were based on data from the entire gender-mixed control group.

**Figure 2:**
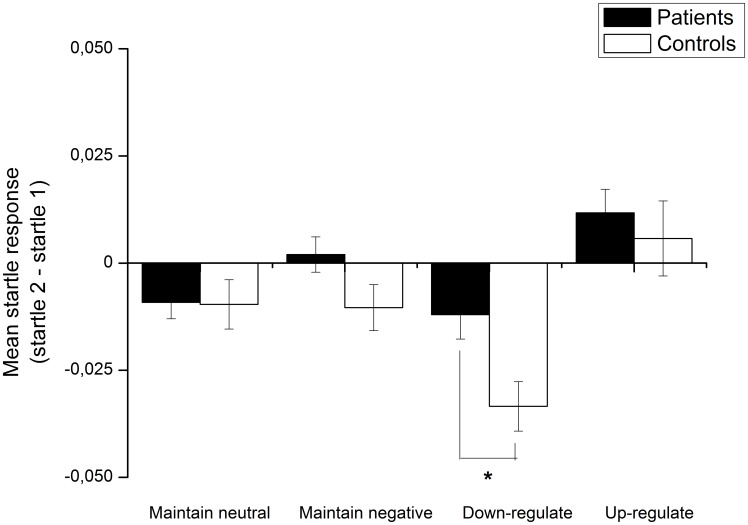
Comparison between burnout patients and controls regarding startle reactions across task instructions. The burnout group displayed overall higher responses when implementing instructions during negative pictures and this pattern was particularly pronounced during down- regulation of negative emotion. Note that the y-axis represents post-instruction response – pre- instruction response; * = p<.05.

**Figure 3:**
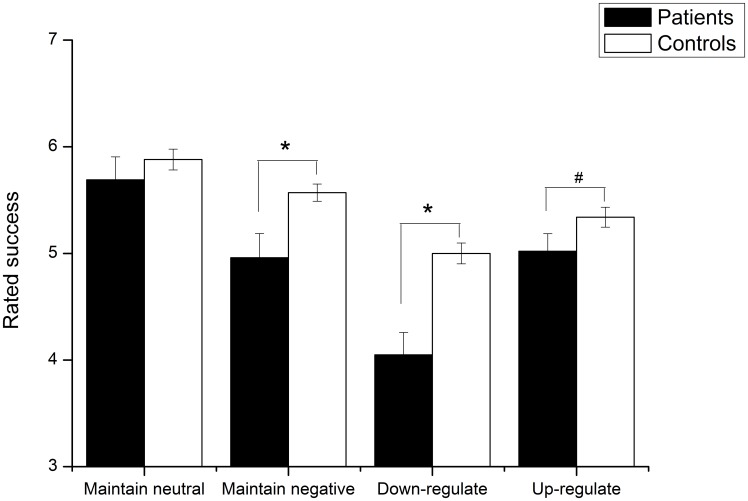
Comparison between burnout patients and controls regarding rated regulation success across task instructions. The burnout group rated themselves as generally less successful at implementing the task instruction after viewing negative pictures. * = p<.05, # = p<.1.

The burnout group and control group did not differ in their initial startle response to negative and neutral pictures (Main effect of Valence: F(1,91) = 39.97, p<.001; Valence×Group interaction: F<1); both groups showed significantly higher startle responses to negative images than to neutral images (burnout: t(31) = 3.87, p = .001; control: t(60) = 5.65, p<.001). However, group differences emerged in the emotional regulation task (see Figure 2). A 2×2 repeated measures ANOVA with Instruction (Down-regulate, Maintain) as the within-subject variable and Group (burnout, control) as the between-subject variable revealed that the burnout population showed overall higher startle responses across instructions (Main effect of Instruction: F(1,91) = 16.32, p<.001 and Group, F(1,91) = 5.55, p = .02). As predicted, follow-up analysis revealed that compared to the controls, the startle response among the burnout patients was significantly higher during negative down-regulation, [t(91) = 2.38, p = .02], but did not reach significance during the negative maintain instruction, [t(91) = 1.55, p = .13]. No significant group difference in startle response was detected during the up-regulate negative condition, [F(1,91) = 6.04, p = .02], or during the maintain neutral condition [t(91) = .06; p = .96]. Lastly, we compared the emotion regulation success ratings of the burnout patients and the controls (see Figure 3). The burnout patients differed from the controls (Instruction×Group: F(2,160) = 4.63, p = .01): the burnout patients had overall lower success ratings after viewing negative images, an effect that was particularly pronounced with regard to being instructed to down-regulate [t(80) = 4.70, p<.001] and maintain negative emotion [t(80) = 3.12, p = .003] but that did not reach significance for up-regulation of negative emotion [t(80) = 1.77; p = .08]. These results parallel those observed with the startle response. Critically, there were no differences between groups with respect to rating after viewing neutral images [t(80) = .94, p = .35].

To test for possible effects of stress (MBI-GS) and depression (MADRS) scores on the ability to down-regulate negative emotion, two separate correlational analyses were run (Pearsson's linear correlation analysis). Bonferroni correction was not employed because significant correlations were hypothesized for both score types. Correlation analyses were carried out using both groups, and also within each separate group. Both higher stress scores (MGI-GS: r = 22, p = .02) and higher depression scores (MADRS: r = . 37, p = .02) were related to a decreased ability to down-regulate negative emotion, as indexed by higher differential startle responses during the negative down-regulation condition. However, neither of these scores correlated with the differential startle responses during down-regulation of negative emotion within the burnout or control groups (both p's>.1).

### Seed region fMRI connectivity

**Figure 4:**
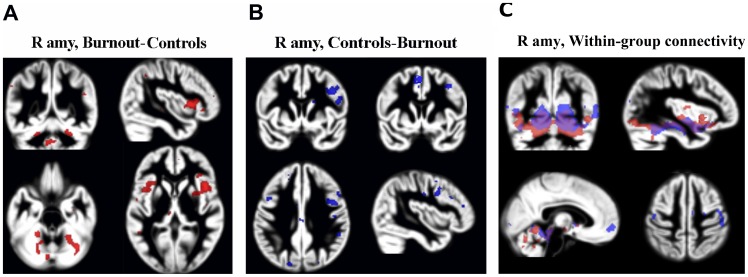
Group difference in resting state functional connectivity from the right amygdala (R amy). Red clusters were calculated from the burnout group - control-group contrast (A), and blue clusters from the reverse contrast (B), (p<0.05 FWE corrected). Clusters are superimposed on the grey matter template (in the MNI space) from the entire study group. (C) Within group connectivity (positive) from the right amygdala. Blue clusters show connectivity clusters in controls, red clusters in the burnout group.

There was a significant difference between stressed subjects and controls with respect to functional connectivity of the right and left amygdala. Stressed subjects showed significantly weaker correlations with clusters in the mPFC, the dorsolateral prefrontal cortex (dlPFC), and the motor cortex, whereas their functional connectivity during resting state with clusters in the cerebellum (vermis cerebelli and the anterior cerebellum in particular) and the insular cortex were stronger than in controls (Table 2, Figure 4a and 4b, clusters calculated at p = .001, cluster level FWE correction at p<.05). These differences were constitutes by differences in positive connectivity and not anticorrelations (please see Figure 4c, which shows within group connectivity patterns).

**Table 2:** Group differences in functional resting state connections from the amygdala.

Region	Z level	Size, cm^3^	Coordinates
**Controls>stressed subjects, R amygdala**			
R PFC+motor cortex	3.8	3.2	44 6 34
**Controls>stressed subjects, L amygdala**			
L mPFC+L dlPFC	4.3	4.8	−48 20 10
			−18 50 22
**Stressed subjects>controls, R amygdala**			
R insular cortex	4.2	4.0	44 14 2
L insular cortex	3.9	3.2	−28 20 6
Cerebellum	4.1	8.0	−22 −22 −28
			26 −32 −26
**Stressed subjects>controls, L amygdala**			
Cerebellum	4.3	3.3	−22 −44 −28

Clusters calculated using voxel threshold at p = 0.001, cluster level FWE correction at p<0.05.

R = right; L = left. The cerebellar clusters covered the anterior cerebellum and the vermis.

### Post hoc analyses

Because the co-variation pattern from the amygdala differed between the two groups of participants, we explored if this could be related to degree of perceived stress or, perhaps, to the MADRS scores. Each subject's MGI-GS and MADRS scores were, therefore, regressed on the individual connectivity maps from the right and left amygdala seed (voxel threshold corresponded to p = .001, FWE cluster correction at p<.05). In addition, given the pivotal role of the mPFC and the ACC in emotional regulation, we tested in the same manner whether the ability to down-regulate negative emotion could be linked to the connectivity between the amygdala and these two regions. Because this analysis was hypothesis based, we employed small volume correction (FWE corrected peak level at p<.05), using a search area defined by a box covering both the ACC and the mPFC, according to Montreal Neurological Institutes (MNI) atlas, the MNI co-ordinates x = −10 to 10; y = 16 to 66; z = 4 to 24.

Only correlational data from the entire study group (thus, without subdivision into burnout subjects and controls) are presented. The subject groups were too limited to allow explorative calculations of possible group differences in the interaction between functional connectivity and emotional regulation or to investigate the respective correlations in each group separately.

**Figure 5:**
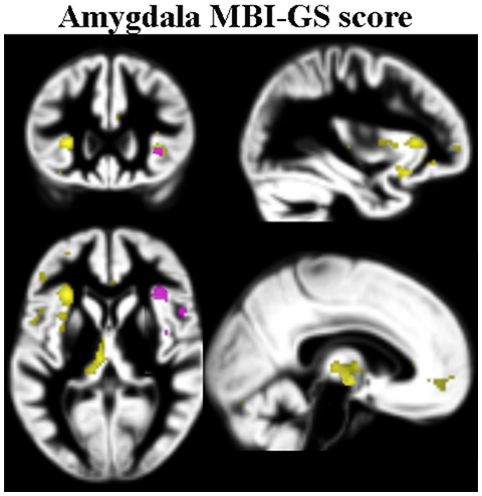
Yellow clusters denote significant interaction between the left amygdala connectivity map and the MBI-GS score merging both groups. Pink clusters denote corresponding clusters from the right amygdala. Clusters calculated at p<0.05 FWE corrected, and superimposed on the grey matter template (in the MNI space) from the entire study group.

The MBI-GS scores (total mean scores) were found to be positively correlated to the functional connectivity between the left amygdala and the insular cortex, and the thalamus (covering a portion of the hypothalamus); the more pronounced the stress perception, the stronger the functional connection was (Table 3, Figure 5). The corresponding analysis involving the right amygdala did not show any significant clusters. There were no negative correlations with the stress scores, nor any interactions with MADRS scores. The ability to down-regulate negative emotion was associated with an increased functional connection between the right amygdala seed and the ACC (the MNI co-ordinate was 6 26 14, z = 3.6, p = .027, FWE corrected, small volume correction).

**Table 3:** Functional resting state connections from the amygdala in relation to stress perception.

Region	Z level	Size, cm^3^	Coordinates
**L amygdala connectivity and MBI-GS**			
L hypothalamus+thalamus	3.9	1.5	−8 −20 6
			−6 −6 6
L insular cortex	4.0	1.0	−32 24 4
**R amygdala connectivity vs MBI-GS**			
R insular cortex	3.7	3.0	36 22 0

The interaction with MBI-GS is calculated at voxel threshold corresponding to p = 0.001, cluster level FWE correction at p<0.05. R = right; L = left. There were no negative covariations from the amygdala seed regions.

## Discussion

The present study represents part of a larger effort to characterize the potential neurobiological underpinnings of occupational burnout, an increasingly reported condition in Western societies. The finding that subjects suffering from chronic occupational stress had an impaired ability to modulate emotion, and weaker functional connectivity between the amygdala and the mPFC (two key structures for orchestrating defensive reactions to environmental threats including stress) supports our previous notion that we are dealing with a condition affecting the limbic system.

Notably, stressed subjects showed higher startle responses specifically during down-regulation of negative emotion, whereas no group difference was detected in the initial response to negative images or in the startle response during up-regulation of negative emotion. Cortisol response during the image presentation was not specifically measured, and it was not possible to objectively verify that the images eliciting negative emotions were perceived as stressful. Such an association has, however, been documented in several previous studies which showed increased cortisol levels as well as increased skin conductance responses during the presentation of negatively valenced IAPS images [59]–[60]. It is, therefore, reasonable to assume that the higher startle response among the burnout group during negative down-regulation reflected an impaired ability to modulate a stressful emotion, although we cannot exclude that this effect was accompanied by differences in attentional resources required to perform the tasks Emotion regulation, as well as stress relies on an intact functional connection between the amygdala, the mPFC, and the ACC [24],[35]. The presently detected functional disconnection between the amygdala and the mPFC in the burnout group as well as the detected interaction between the ability to down-modulate negative emotion and the amygdala–ACC connection confirm this notion. These findings are in line with our previous observation based on PET measurements of resting state connectivity [22], although the methodology and participant sample were different. They are also in accordance with the reduction of gray matter volume in the dlPFC and ACC and the cortical thinning of the mPFC observed in subjects suffering from occupational stress [21],[23]. Taken together, these data support the postulation that stress-processing limbic networks are affected in subjects suffering from occupational burnout.

The cluster showing impaired functional connectivity from the amygdala in the burnout population comprised portions of the motor cortex, which may be due to the fact that our groups were gender mixed. In men there are strong connectivity between the amygdala and motor cortex, while women have strong connectivity between the amygdala and both the mPFC and ACC [61]. Thus, assuming that the amygdala–frontal lobe connectivity were impaired in both sexes among the burnout population, it is not surprising that a cluster encompassing both the mPFC and the motor cortex was observed when comparing the entire control group with the entire burnout group.

The MGI-GS scores interacted with functional connectivity between the amygdala, the thalamus, and a minor portion of the hypothalamus (Figure 5); thus, the more stressed the subject, the stronger the connectivity was. This finding is congruent with the well-established finding that stress leads to the activation of the HPA via amygdala connectivity with the paraventricular hypothalamic nuclei. It also fits well with the notion that the insular cortex relays stress signals from the amygdala to the autonomous nervous system. The observance of a stress-related enhancement of the amygdala–insular cortex connection is in accordance with previous findings for other stress conditions [62].

The enhanced connectivity observed between the amygdala and the cerebellum in the stressed group was, on the other hand, not directly expected. Nevertheless, there are several previous reports suggesting that cerebellum may have a modulatory role in the processing of psychosocial stress. The amygdala relays the emotional salience of incoming signals to the rest of the brain, and via cholinergic connections to the pontine nuclei and the cerebellum, the neuronal traffic from the amygdala leads to increased arousal [24]–[25]. The cerebellum is part of the amygdala's resting state connectivity network [63], and via inhibitory (GABAergic) output from Purkinje, the excitation of the amygdala is modulated by the cerebellum. Via this ability to modulate the excitation of the amygdala the cerebellum is involved in the processing of emotion, and potentially also the psychosocial stress. Interestingly, the cerebellum has dense glucocorticoid binding sites as well as reciprocal monosynaptic connections to the hypothalamus, that provide a biological substrate for the regulation of HPA, and the stress response. Animal experiments show an enlargement of Purkinje cell spines in response to corticotropin-releasing factor [64]. The involvement of the cerebellum in stress is also indicated by reports about a reduction in the cerebellar volume in patients with PTSD [65]–[67]. Thus, although there have not been any comparable previous studies showing stress- related increases in the functional connectivity between the amygdala and cerebellum, one may speculate that, in our burnout population, a compensatory enhancement of the modulatory pathway from the cerebellum could have occurred due to weakened amygdala–mPFC connectivity.

### Methodological limitations and future directions

The amygdala seed covered the entire amygdala except for its most medial portion, which was excluded because of the signal loss in the fMRI images; thus, no differentiation could be made between the basomedial and dorsolateral nuclei. In previous studies of stress with resting state fMRI, primarily carried out in patients with PTSD, it has been reported that amygdala–mPFC (and also the amygdala-ACC) connectivity is decreased when seeding from the entire amygdala [68]–[69]. However, when separating the medial and lateral portions of the amygdala, Brown et al. found elevated connectivity between the basomedial-amygdala and the insular and dorsomedial PFC in PTDS patients, whereas the connection between the lateral amygdala and the inferior frontal cortex was stronger in controls [70]. It is, thus, possible that the results with respect to amygdala connectivity would be slightly different if the two major portions of the amygdala were separated. In this initial study, our priority was, however, to minimize the noise in the seed ROI. Also, there was no primary hypothesis that the mesial and lateral portions of the amygdala would be affected differently in the burnout population.

The relatively small size of the subject sample did not allow us to test for possible gender-related differences in our results among the burnout group. This important issue will be investigated in a separate study. The groups were, however, matched with respect to gender distribution.

One important question worth discussing is whether and to what extent the present findings could reflect depression. For several reasons we find this to be unlikely. First of all, none of the stressed subjects reported dysthymia, and none were judged to be depressed according to the psychiatrist in charge and the SCID-MINI ratings. Although the depression scores were significantly higher among the stressed group, for those subjects who had high MADRS scores, the only items that contributed to these scores were anxiety and poor sleep, which does not necessarily imply depression. Higher MADRS scores were related to a decreased ability to down-regulate negative emotion, but these scores covaried highly with the stress scores, and did not moderate the ability to down-regulate negative emotion within the burnout group. While the MGI-GS scores interacted with the amygdala connectivity with the insular cortex and the thalamus, no such interaction was detected with the MADRS scores, not even when employing small volume correction. Finally, the cortisol levels were normal in our stressed subjects, whereas they have been found to be high in a large portion of patients with genuine depression [71]. We recently also found that women suffering from chronic occupational stress had an elevated reaction to allopregnanolone [20] which differed from the diminished allopregnanolone response that has been observed among depressed women [72]. Emotional reactions to chronic stress and major depression may, thus, represent separate constructs. They share, however, certain symptoms perhaps due to the affection of similar limbic networks. Indeed, the higher MADRS scores among the stressed subjects who were not diagnosed as depressed could be an effect of this comorbidity. Stress may lead to depression, and the impaired ability to specifically down-regulate negative emotions that was demonstrated in the present study may by itself render stressed individuals more prone to depressive thoughts and explain the comorbidity between the two conditions. Because the study was cross-sectional, it is difficult to know whether the detected changes represent effects of stress or of a pre-existing condition that could have rendered the brain more vulnerable to the development of pathological stress responses and reduced the ability to modulate emotion.

## Conclusion

In subjects suffering from chronic emotional stress, there seems to be a dysregulation of the emotion- and stress-processing networks, which prevents the restoration of internal homeostasis in response to negative emotional stress. An impairment of the ability to down-regulate negative emotions in subjects suffering from occupational stress may render them more vulnerable to depressive symptoms. This finding needs to be further explored, as it may potentially explain the link between stress and psychological ill health.

## References

[1] Eisler RM, Polak PR (1971) Social stress and psychiatric disorder. J Nerv Ment Dis 153: 227–233. 5114083 10.1097/00005053-197110000-00001

[2] Weber K, Miller GA, Schupp HT, Borgelt J, Awiszus B, et al (2009) Early life stress and psychiatric disorder modulate cortical responses to affective stimuli. Psychophysiology 46: 1234–1243. 19674395 10.1111/j.1469-8986.2009.00871.x

[3] Kalia M (2002) Assessing the economic impact of stress–the modern day hidden epidemic. Metabolism: clinical and experimental 51: 49–53. 12040542 10.1053/meta.2002.33193

[4] Ahola K, Honkonen T, Isometsa E, Kalimo R, Nykyri E, et al (2006) Burnout in the general population. Results from the Finnish Health 2000 Study. Soc Psychiatry Psychiatr Epidemiol 41: 11–17. 16341826 10.1007/s00127-005-0011-5

[5] Copertaro A, Barbaresi M, Tarsitani L, Battisti F, Baldassari M, et al (2007) [Fast stress evaluation in nurses]. Giornale italiano di medicina del lavoro ed ergonomia 29: 350–352. 18409720

[6] Copertaro A, Bracci M, Amati M, Mocchegiani E, Barbaresi M, et al (2010) [Biological risk and health care workers: analysis of the effects of work chronobiology on the immune system]. Med Lav 101: 427–436. 21141454

[7] Fernandez Torres B, Roldan Perez LM, Guerra Velez A, Roldan Rodriguez T, Gutierrez Guillen A, et al (2006) [Prevalence of burnout among anesthesiologists at Hospital Universitario Virgen Macarena de Sevilla]. Rev Esp Anestesiol Reanim 53: 359–362. 16910143

[8] Rydmark I, Wahlberg K, Ghatan PH, Modell S, Nygren A, et al (2006) Neuroendocrine, cognitive and structural imaging characteristics of women on longterm sickleave with job stress-induced depression. Biological psychiatry 60: 867–873. 16934773 10.1016/j.biopsych.2006.04.029

[9] Fries E, Dettenborn L, Kirschbaum C (2009) The cortisol awakening response (CAR): facts and future directions. Int J Psychophysiol 72: 67–73. 18854200 10.1016/j.ijpsycho.2008.03.014

[10] Langelaan S, Bakker AB, Schaufeli WB, van Rhenen W, van Doornen LJ (2007) Is burnout related to allostatic load? Int J Behav Med 14: 213–221. 18001236 10.1007/BF03002995

[11] Mommersteeg PM, Heijne CJ, Verbraak MJ, van Doornen LJ (2006a) Clinical burnout is not reflected in the cortisol awakening response, the day-curve or the response to a low-dose dexamethasone suppression test. Psychoneuroendocrinology 31: 216–225. 16150550 10.1016/j.psyneuen.2005.07.003

[12] Chida Y, Steptoe A (2009) Cortisol awakening response and psychosocial factors: a systematic review and meta-analysis. Biol Psychol 80: 265–278. 19022335 10.1016/j.biopsycho.2008.10.004

[13] Moch SL, Panz VR, Joffe BI, Havlik I, Moch JD (2003) Longitudinal changes in pituitary- adrenal hormones in South African women with burnout. Endocrine 21: 267–272. 14515012 10.1385/ENDO:21:3:267

[14] Mommersteeg PM, Keijsers GP, Heijnen CJ, Verbraak MJ, van Doornen LJ (2006b) Cortisol deviations in people with burnout before and after psychotherapy: a pilot study. Health Psychol 25: 243–248. 16569117 10.1037/0278-6133.25.2.243

[15] Pruessner JC, Hellhammer DH, Kirschbaum C (1999) Burnout, perceived stress, and cortisol responses to awakening. Psychosom Med 61: 197–204. 10204973 10.1097/00006842-199903000-00012

[16] Grossi G, Perski A, Ekstedt M, Johansson T, Lindstrom M, et al (2005) The morning salivary cortisol response in burnout. J Psychosom Res 59: 103–111. 16186006 10.1016/j.jpsychores.2005.02.009

[17] Grossi G, Perski A, Evengard B, Blomkvist V, Orth-Gomer K (2003) Physiological correlates of burnout among women. J Psychosom Res 55: 309–316. 14507541 10.1016/s0022-3999(02)00633-5

[18] Melamed S, Ugarten U, Shirom A, Kahana L, Lerman Y, Froom P (1999) Chronic burnout, somatic arousal and elevated salivary cortisol levels. J Psychosom Res 46: 591–598. 10454175 10.1016/s0022-3999(99)00007-0

[19] Sandstrom A, Rhodin IN, Lundberg M, Olsson T, Nyberg L (2005) Impaired cognitive performance in patients with chronic burnout syndrome. Biological psychology 69: 271–279. 15925030 10.1016/j.biopsycho.2004.08.003

[20] Backstrom T, Bixo M, Nyberg S, Savic I (2013) Increased neurosteroid sensitivity–an explanation to symptoms associated with chronic work related stress in women? Psychoneuroendocrinology 38: 1078–1089. 23177572 10.1016/j.psyneuen.2012.10.014

[21] Blix E, Perski A, Berglund H, Savic I (2013) Long-term occupational stress is associated with regional reductions in brain tissue volumes. PLoS ONE 8: e64065. 23776438 10.1371/journal.pone.0064065 PMC3679112

[22] Jovanovic H, Perski A, Berglund H, Savic I (2011) Chronic stress is linked to 5-HT(1A) receptor changes and functional disintegration of the limbic networks. Neuroimage 55: 1178–1188. 21211567 10.1016/j.neuroimage.2010.12.060

[23] Savic I Structural changes of the brain in relation to occupational stress. Cereb Cortex 2013 Dec 18. [Epub ahead of print]. 10.1093/cercor/bht348 24352030

[24] LeDoux JE (2000) Emotion circuits in the brain. Annual review of neuroscience 23: 155–184. 10.1146/annurev.neuro.23.1.155 10845062

[25] Phillips ML, Drevets WC, Rauch SL, Lane R (2003) Neurobiology of emotion perception I: The neural basis of normal emotion perception. Biological Psychiatry 54: 504–514. 12946879 10.1016/s0006-3223(03)00168-9

[26] Egner T, Etkin A, Gale S, Hirsch J (2008) Dissociable neural systems resolve conflict from emotional versus nonemotional distracters. Cereb Cortex 18: 1475–1484. 17940084 10.1093/cercor/bhm179

[27] Etkin A, Egner T, Peraza DM, Kandel ER, Hirsch J (2006) Resolving emotional conflict: A role for the rostral anterior cingulate cortex in modulating activity in the amygdala. Neuron 51: 871–882. 16982430 10.1016/j.neuron.2006.07.029

[28] Gianaros PJ, Sheu LK, Matthews KA, Jennings JR, Manuck SB, et al (2008) Individual differences in stressor-evoked blood pressure reactivity vary with activation, volume, and functional connectivity of the amygdala. J Neurosc 28: 990–999. 10.1523/JNEUROSCI.3606-07.2008 PMC2526972 18216206

[29] Pezawas L, Meyer-Lindenberg A, Drabant EM, Verchinski BA, Munoz KE, et al (2005) 5-HTTLPR polymorphism impacts human cingulate-amygdala interactions: a genetic susceptibility mechanism for depression. Nature neuroscience 8: 828–834. 15880108 10.1038/nn1463

[30] Wager TD, Davidson ML, Hughes BL, Lindquist MA, Ochsner KN (2008) Prefrontal-subcortical pathways mediating successful emotion regulation. Neuron 59: 1037–1050. 18817740 10.1016/j.neuron.2008.09.006 PMC2742320

[31] Goldin PR, McRae K, Ramel W, Gross JJ (2008) The neural bases of emotion regulation: Reappraisal and suppression of negative emotion. Biological Psychiatry 63: 577–586. 17888411 10.1016/j.biopsych.2007.05.031 PMC2483789

[32] Golkar A, Lonsdorf TB, Olsson A, Lindstrom KM, Berrebi J, et al (2012) Distinct contributions of the dorsolateral prefrontal and orbitofrontal cortex during emotion regulation. PLoS ONE 7: e48107. 23144849 10.1371/journal.pone.0048107 PMC3492343

[33] Koenigsberg HW, Fan J, Ochsner KN, Liu X, Guise K, et al (2010) Neural correlates of using distancing to regulate emotional responses to social situations. Neuropsychologia 48: 1813–1822. 20226799 10.1016/j.neuropsychologia.2010.03.002 PMC2905649

[34] Levesque J, Eugene F, Joanette Y, Paquette V, Mensour B, et al (2003) Neural circuitry underlying voluntary suppression of sadness. Biological Psychiatry 53: 502–510. 12644355 10.1016/s0006-3223(02)01817-6

[35] Ochsner KN, Bunge SA, Gross JJ, Gabrieli JDE (2002) Rethinking feelings: An fMRI study of the cognitive regulation of emotion. Journal of Cognitive Neuroscience 14: 1215–1229. 12495527 10.1162/089892902760807212

[36] Ochsner KN, Ray RD, Cooper JC, Robertson ER, Chopra S, et al (2004) For better or for worse: neural systems supporting the cognitive down- and up-regulation of negative emotion. Neuroimage 23: 483–499. 15488398 10.1016/j.neuroimage.2004.06.030

[37] Raio CM, Orederu TA, Palazzolo L, Shurick AA, Phelps EA (2013) Cognitive emotion regulation fails the stress test. Proc Natl Acad Sci U S A 110: 15139–15144. 23980142 10.1073/pnas.1305706110 PMC3773739

[38] Davis M, Parisi T, Gendelman DS, Tischler M, Kehne JH (1982) Habituation and sensitization of startle reflexes elicited electrically from the brain-stem. Science 218: 688–690. 7134967 10.1126/science.7134967

[39] Yeomans JS, Frankland PW (1995) The acoustic startle reflex: Neurons and connections. Brain research reviews 21: 301–314. 8806018 10.1016/0165-0173(96)00004-5

[40] Lang PJ, Bradley MM, Cuthbert BN (1990) Emotion, attention, and the startle reflex. Psychological Review 97: 377–395. 2200076

[41] Bradley MM, Cuthbert BN, Lang PJ (1990) Startle reflex modification - emotion or attention. Psychophysiology 27: 513–522. 2274614 10.1111/j.1469-8986.1990.tb01966.x

[42] Dillon DG, LaBar KS (2005) Startle modulation during conscious emotion regulation is arousal- dependent. Behav Neurosci 119: 1118–1124. 16187839 10.1037/0735-7044.119.4.1118

[43] Jackson DC, Malmstadt JR, Larson CL, Davidson RJ (2000) Suppression and enhancement of emotional responses to unpleasant pictures. Psychophysiology 37: 515–522. 10934910

[44] Oldfield RC (1971) The assessment and analysis of handedness: the Edinburgh inventory. Neuropsychologia 9: 97–113. 5146491 10.1016/0028-3932(71)90067-4

[45] Schaufeli WB, Van Dierendonck D (1995) A cautionary note about the cross-national and clinical validity of cut-off points for the Maslach Burnout Inventory. Psychological reports 76: 1083–1090. 7480470 10.2466/pr0.1995.76.3c.1083

[46] Stenlund T, Ahlgren C, Lindahl B, Burell G, Knutsson A (2007) Patients with burnout in relation to gender and a general population. Scandinavian journal of public health 35: 516–523. 17852977 10.1080/14034940701271874

[47] Deykin EY, Keane TM, Kaloupek D, Fincke G, Rothendler J, et al (2001) Posttraumatic stress disorder and the use of health services. Psychosom Med 63: 835–841. 11573033 10.1097/00006842-200109000-00018

[48] Holmes TH, Rahe RH (1967) The Social Readjustment Rating Scale. J Psychosom Res 11: 213–218. 6059863 10.1016/0022-3999(67)90010-4

[49] Sheehan DV, Lecrubier Y, Sheehan KH, Amorim P, Janavs J (1998) The Mini-International Neuropsychiatric Interview (M.I.N.I.): the development and validation of a structured diagnostic psychiatric interview for DSM-IV and ICD-10. J Clin Psychiatry 59 Suppl 20: 22–33 quiz 34–57. 9881538

[50] Montgomery S, Asberg M, Traskman L, Montgomery D (1978) Cross cultural studies on the use of CPRS in English and Swedish depressed patients. Acta Psychiatrica Scandinavica Suppl 271: 33–37. 10.1111/j.1600-0447.1978.tb02359.x 277056

[51] Karlamangla AS, Friedman EM, Seeman TE, Stawksi RS, Almeida DM (2013) Daytime trajectories of cortisol: Demographic and socioeconomic differences-Findings from the National Study of Daily Experiences. Psychoneuroendocrinology 38: 2585–2597. 23831263 10.1016/j.psyneuen.2013.06.010 PMC3812359

[52] Galbois A, Rudler M, Massard J, Fulla Y, Bennani A, et al (2010) Assessment of adrenal function in cirrhotic patients: salivary cortisol should be preferred. J Hepatol 52: 839–845. 20385427 10.1016/j.jhep.2010.01.026

[53] Lang PJ, Bradley MM, Cuthbert BN (1997) International Affective Picture System (IAPS): Technical manual and affective ratings. Gainesville, FL: NIMH Center for the Study of Emotion and Attention, University of Florida.

[54] Jackson DC, Mueller CJ, Dolski I, Dalton KM, Nitschke JB, et al (2003) Now you feel it, now you don't: Frontal brain electrical asymmetry and individual differences in emotion regulation. Psychological science 14: 612–617. 14629694 10.1046/j.0956-7976.2003.psci_1473.x

[55] Golkar A, Bellander M, Ohman A (2013) Temporal properties of fear extinction–does time matter? Behav Neurosci 127: 59–69. 23231494 10.1037/a0030892

[56] Weike AI, Schupp HT, Hamm AO (2007) Fear acquisition requires awareness in trace but not delay conditioning. Psychophysiology 44: 170–180. 17241153 10.1111/j.1469-8986.2006.00469.x

[57] Biswal B, Yetkin FZ, Haughton VM, Hyde JS (1995) Functional connectivity in the motor cortex of resting human brain using echo-planar MRI. Magn Reson Med 34: 537–541. 8524021 10.1002/mrm.1910340409

[58] Weissenbacher A, Kasess C, Gerstl F, Lanzenberger R, Moser E, et al (2009) Correlations and anticorrelations in resting-state functional connectivity MRI: a quantitative comparison of preprocessing strategies. Neuroimage 47: 1408–1416. 19442749 10.1016/j.neuroimage.2009.05.005

[59] Bradley MM, Codispoti M, Cuthbert BN, Lang PJ (2001) Emotion and Motivation I: Defensive and Appetitive Reactions in Picture Processing. Emotion 1: 276–298. 12934687

[60] Gerra G, Somaini L, Manfredini M, Raggi MA, Saracino MA, et al (2014) Dysregulated responses to emotions among abstinent heroin users: Correlation with childhood neglect and addiction severity,. Progress in Neuro-Psychopharmacology and Biological Psychiatry 48: 220–228. 24161666 10.1016/j.pnpbp.2013.10.011

[61] Savic I, Lindstrom P (2008) PET and MRI show differences in cerebral asymmetry and functional connectivity between homo- and heterosexual subjects. Proc Natl Acad Sci U S A 105: 9403–9408. 18559854 10.1073/pnas.0801566105 PMC2453705

[62] Hong JY, Kilpatrick LA, Labus J, Gupta A, Jiang Z, et al (2013) Patients with chronic visceral pain show sex-related alterations in intrinsic oscillations of the resting brain. J Neurosci 33: 11994–12002. 23864686 10.1523/JNEUROSCI.5733-12.2013 PMC3713732

[63] Sang L, Qin W, Liu Y, Han W, Zhang Y, et al (2012) Resting-state functional connectivity of the vermal and hemispheric subregions of the cerebellum with both the cerebral cortical networks and sub.cortical structures. Neuroimage 61: 1213–1225. 22525876 10.1016/j.neuroimage.2012.04.011

[64] Gounko NV, Swinny JD, Kalicharan D, Jafari S, Corteen N, et al (2013) Corticotropin-releasing factor and urocortin regulate spine and synapse formation: structural basis for stress-induced neuronal remodeling and pathology. Molecular psychiatry 18: 86–92. 22547117 10.1038/mp.2012.43

[65] Anderson CM, Teicher MH, Polcari A, Renshaw PF (2002) Abnormal T2 relaxation time in the cerebellar vermis of adults sexually abused in childhood: potential role of the vermis in stress-enhanced risk for drug abuse. Psychoneuroendocrinology 27: 231–244. 11750781 10.1016/s0306-4530(01)00047-6

[66] De Bellis MD, Kuchibhatla M (2006) Cerebellar volumes in pediatric maltreatment-related posttraumatic stress disorder. Biol Psychiatry 60: 697–703. 16934769 10.1016/j.biopsych.2006.04.035

[67] Baldacara L, Jackowski AP, Schoedl A, Pupo M, Andreoli SB, et al (2011) Reduced cerebellar left hemisphere and vermal volume in adults with PTSD from a community sample. J Psychiatr Res 45: 1627–1633. 21824628 10.1016/j.jpsychires.2011.07.013

[68] Rabinak CA, Angstadt M, Welsh RC, Kenndy AE, Lyubkin M, et al (2011) Altered amygdala resting-state functional connectivity in post-traumatic stress disorder. Frontiers in psychiatry 2: 62. 22102841 10.3389/fpsyt.2011.00062 PMC3214721

[69] Sripada RK, King AP, Garfinkel SN, Wang X, Sripada CS, et al (2012) Altered resting-state amygdala functional connectivity in men with posttraumatic stress disorder. J Psychiatry Neurosci 37: 241–249. 22313617 10.1503/jpn.110069 PMC3380095

[70] Brown VM, Labar KS, Haswell CC, Gold AL (2014) Mid-Atlantic MIRECC Workgroup, (2014) et al Altered resting-state functional connectivity of basolateral and centromedial amygdala complexes in posttraumatic stress disorder. Neuropsychopharmacology 39: 351–359. 23929546 10.1038/npp.2013.197 PMC3870774

[71] Kasckow JW, Baker D, Geracioti TD Jr (2001) Corticotropin-releasing hormone in depression and post-traumatic stress disorder. Peptides 22: 845–851. 11337099 10.1016/s0196-9781(01)00399-0

[72] Girdler SS, Klatzkin R (2007) Neurosteroids in the context of stress: implications for depressive disorders. Pharmacology & therapeutics 116: 125–139. 17597217 10.1016/j.pharmthera.2007.05.006 PMC2650267

